# Supplementary material for: Mapping mutations to the SARS-CoV-2 RBD that escape binding by different classes of antibodies
Source: Nat Commun. 2021 Jul 7;12:4196. doi: 10.1038/s41467-021-24435-8 (PMC8263750; doi:10.1038/s41467-021-24435-8)
Supplement: Supplementary file 1 — Supplementary Information [file 41467_2021_24435_MOESM1_ESM.pdf]

**Mapping mutations to the SARS-CoV-2 RBD that escape binding by different classes of antibodies**

Allison J. Greaney[1,2], Tyler N. Starr[1,3], Christopher O. Barnes[4], Yiska Weisblum[5], Fabian Schmidt[5], Marina Caskey[6], Christian Gaebler[6], Alice Cho[6], Marianna Agudelo[6], Shlomo Finkin[6], Zijun Wang[6], Daniel Poston[5], Frauke Muecksch[5], Theodora Hatziioannou[5], Paul D. Bieniasz[3,5], Davide F. Robbiani[6,7], Michel C. Nussenzweig[3,6], Pamela J. Bjorkman[4], Jesse D. Bloom[1,3,#]

**Supplementary Information:**
**Figures S1–S6**
**Supplementary Table 1**

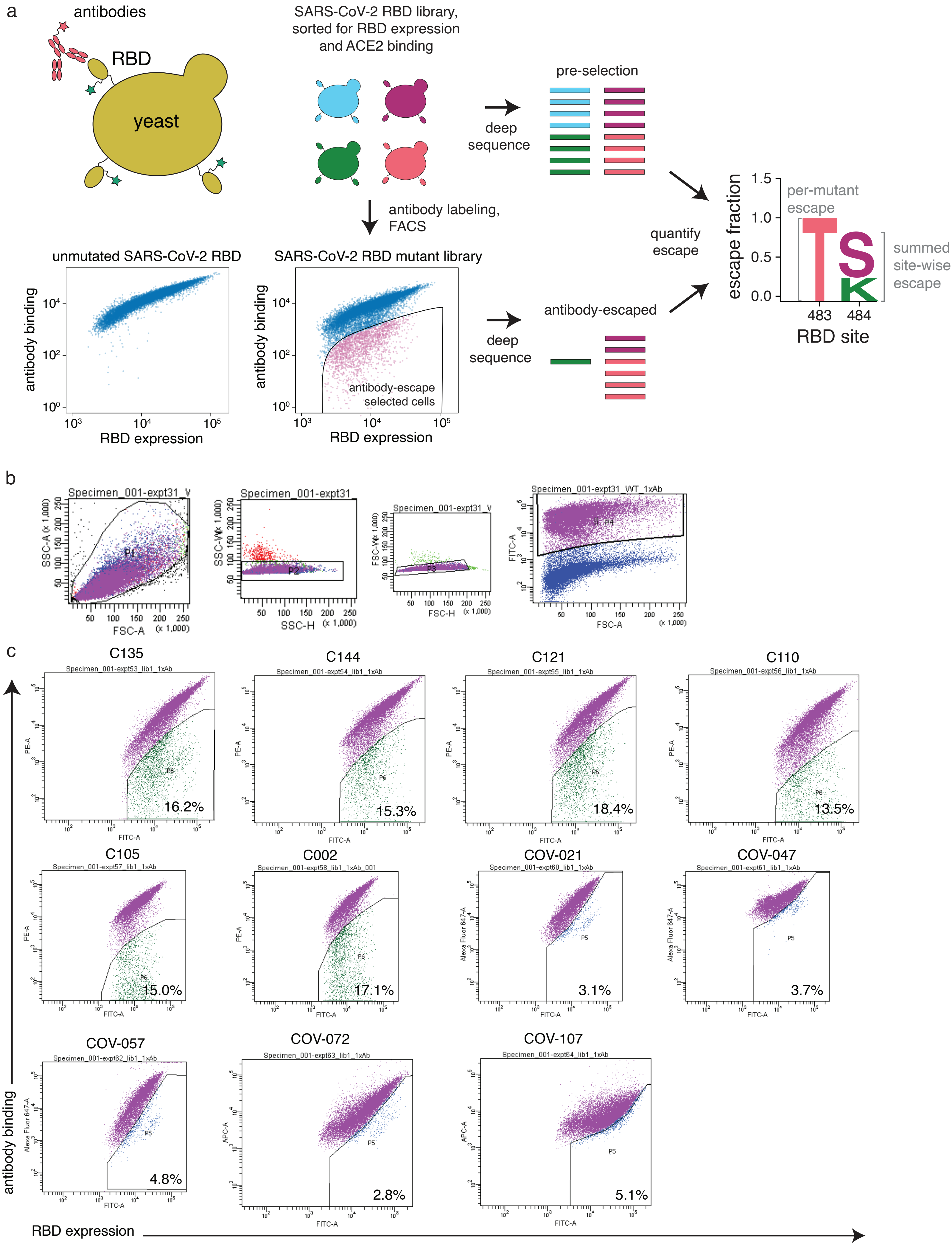
a
antibodies
RBD
yeast
SARS-CoV-2 RBD library, sorted for RBD expression and ACE2 binding
pre-selection
deep sequence
antibody labeling, FACS
unmutated SARS-CoV-2 RBD
SARS-CoV-2 RBD mutant library
antibody binding
RBD expression
antibody-escape selected cells
antibody-escaped
quantify escape
escape fraction
per-mutant escape
summed site-wise escape
483
484
RBD site
b
Specimen_001-expt31_WT_1xAb
SSC-A
FSC-A
SSC-W
SSC-H
FSC-W
FSC-H
FITC-A
P1
P2
P3
P4
c
C135
16.2%
C144
15.3%
C121
18.4%
C110
13.5%
C105
15.0%
C002
17.1%
COV-021
3.1%
COV-047
3.7%
COV-057
4.8%
COV-072
2.8%
COV-107
5.1%
PE-A
Alexa Fluor 647-A
APC-A
P5
P6
antibody binding
RBD expression

**Figure S1**. **Approach for mapping RBD mutations that reduce binding by monoclonal antibodies or polyclonal plasma, related to Figure 1. (A)** The RBD is expressed on the surface of yeast. Flow cytometry is used to quantify both RBD expression (via a C-terminal MYC tag) and antibody binding to the RBD protein expressed on the surface of each yeast cell. A library of yeast expressing different RBD mutants were incubated with antibodies or plasma and binding was detected using a IgG or IgA+IgG+IgM secondary antibody for monoclonal antibodies or polyclonal plasma, respectively. We then used FACS to enrich for cells expressing RBD that bound reduced levels of antibody, and deep sequencing to quantify the frequency of each mutation in the initial and antibody-escape cell populations. We quantified the effect of each mutation as the "escape fraction," which represents the fraction of cells expressing RBD with that mutation that fell in the antibody escape FACS bin. Escape fractions are represented in logo plots, with the height of each letter proportional to the effect of that amino-acid mutation on antibody binding. The site-level escape metric is the sum of the escape fractions of all mutations at a site. Note that both experimental and computational filtering steps were used to remove RBD mutants that were misfolded or completely unable to bind the ACE2 receptor (see **Methods**). **(B)** Representative plots of nested FACS gating strategy used for all plasma selection experiments to select for single cells (SSC-A vs. FSC-A, SSC-W vs. SSC-H, and FSC-W vs. FSC-H) that also express RBD (FITC-A vs. FSC-A). **(C)** FACS gating strategy for one of two independent libraries to select cells expressing RBD mutants with reduced binding by monoclonal antibodies or polyclonal plasma (cells in blue). Gates were set manually during sorting. Different strategies were used for monoclonal antibodies vs. polyclonal plasma. For monoclonal antibodies, selection gates were set to capture up to 95% of yeast cells expressing unmutated RBD, stained with an antibody concentration 100x lower than that used for library staining. For polyclonal plasma, selection gates were set to capture 3-6% of the RBD+ library. The same gate was set for both independent libraries stained with each plasma, and the FACS scatter plots looked qualitatively similar between the two libraries. For information on the fraction of library cells that fall into each selection gate, see **Supplementary Table 1**.

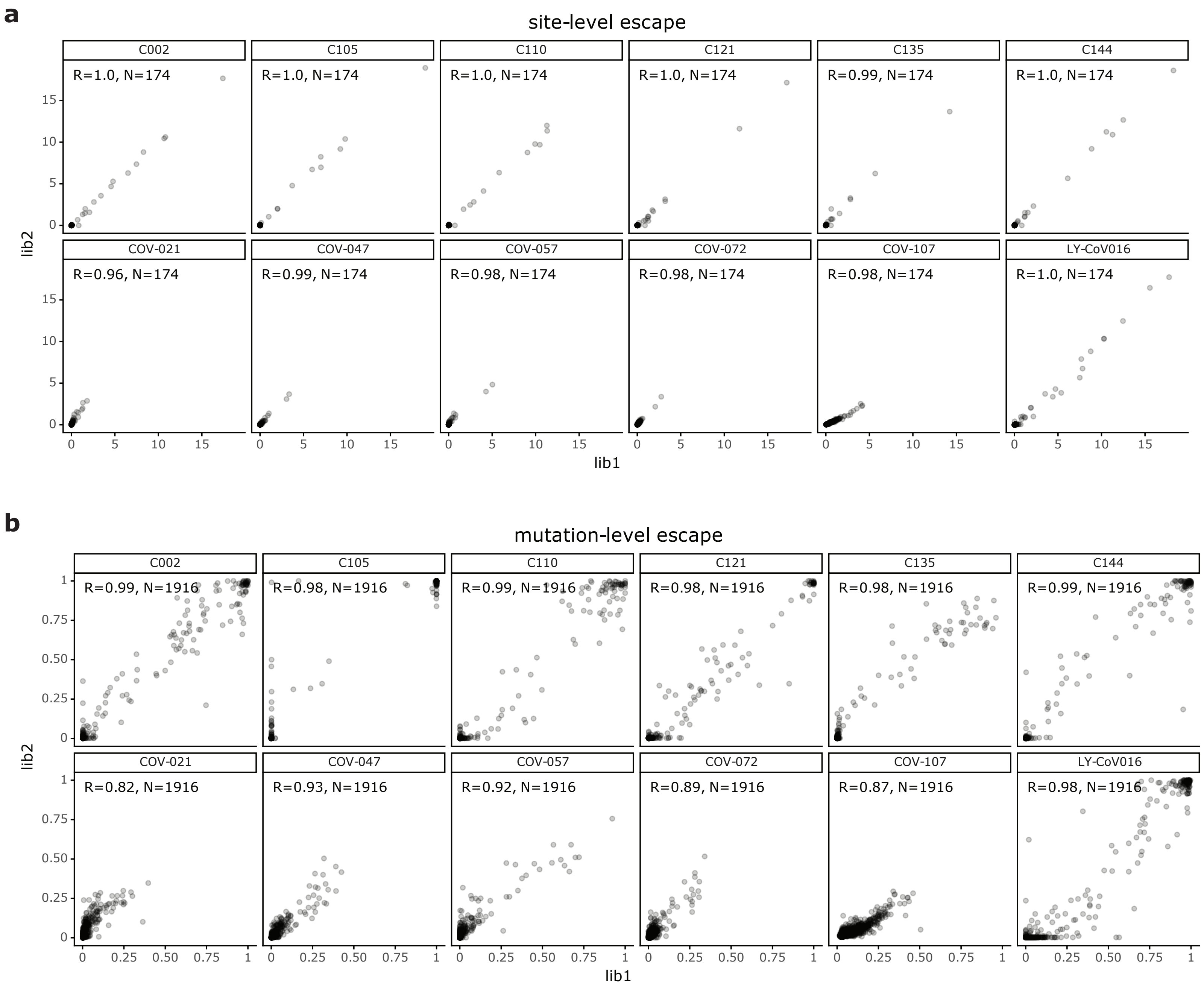


**Figure S2. Correlations between replicates for site- and mutation-level escape metrics, related to Figure 1. (A)** Correlation plots of site-level escape for each of the two independent RBD mutant libraries for each antibody or plasma. Each point represents one site in the RBD. **(B)** Correlation plots of mutation-level escape for each of the two independent RBD mutant libraries for each antibody or plasma. In this plot, each point represents a different mutation.

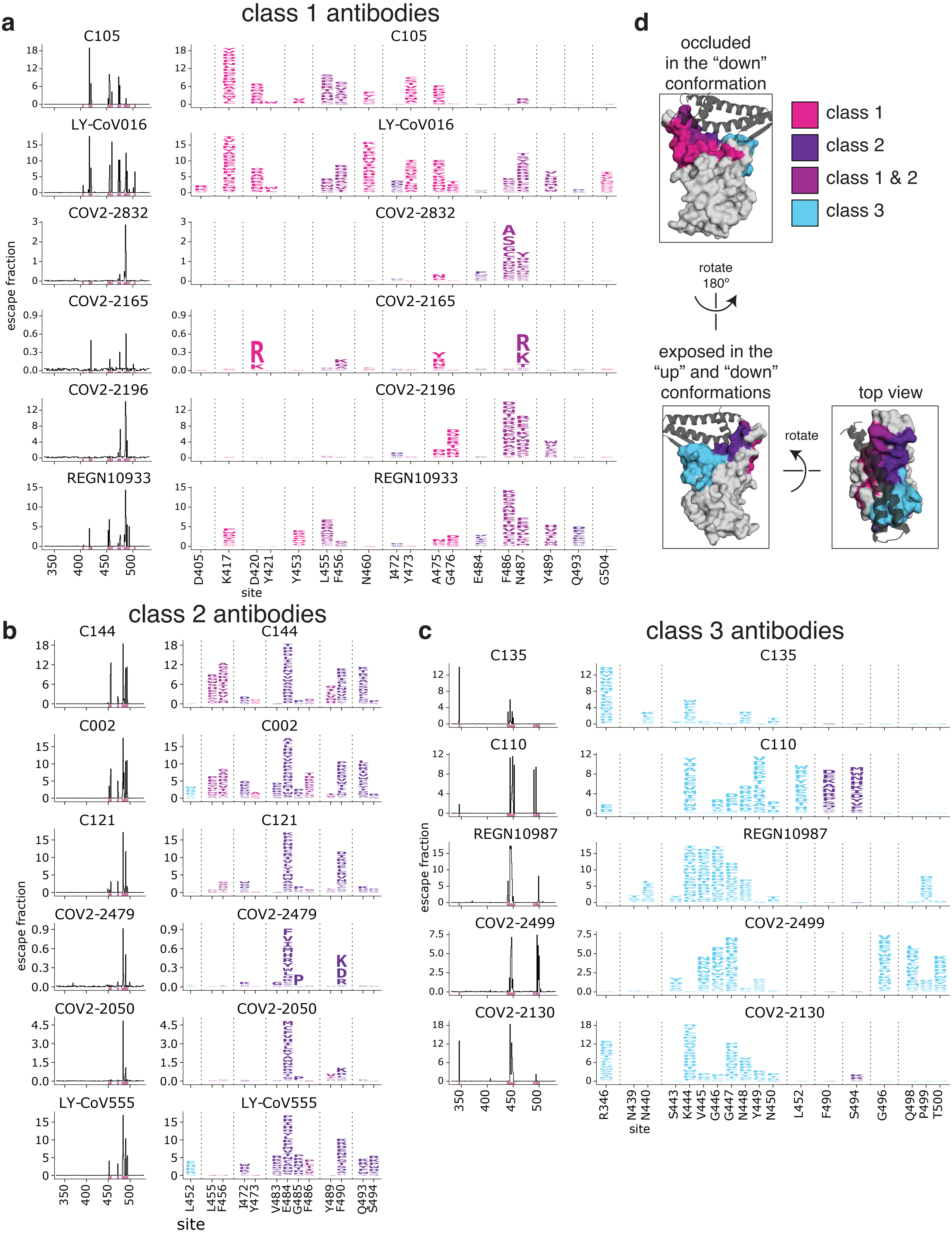
a
class 1 antibodies
C105
LY-CoV016
COV2-2832
COV2-2165
COV2-2196
REGN10933
escape fraction
site
D405
K417
D420
Y421
Y453
L455
F456
N460
I472
Y473
A475
G476
E484
F486
N487
Y489
Q493
G504
d
occluded in the "down" conformation
class 1
class 2
class 1 & 2
class 3
rotate 180°
exposed in the "up" and "down" conformations
top view
rotate
b
class 2 antibodies
C144
C002
C121
COV2-2479
COV2-2050
LY-CoV555
escape fraction
site
L452
L455
F456
I472
Y473
V483
E484
G485
F486
Y489
F490
Q493
S494
c
class 3 antibodies
C135
C110
REGN10987
COV2-2499
COV2-2130
escape fraction
site
R346
N439
N440
S443
K444
V445
G446
G447
N448
Y449
N450
L452
F490
S494
G496
Q498
P499
T500

**Figure S3. Escape maps for class 1, 2, or 3 antibodies we have profiled here or in previous studies, related to Figure 1.** Escape maps for **(A)** class 1, **(B)** class 2, or **(C)** class 3 antibodies shown in **Figure 1C**. All escape maps were previously generated[30,33,35,36] except for C105, C144, C002, C121, C135, and C110 which are new to this study. Different sets of key sites are shown for each of the three antibody classes (see **Methods**). Sites are colored by RBD epitope as in **Figure 1,** also shown in panel **(D)**. For the escape maps of class 4 antibodies shown in **Figure 1C**, see Greaney et al. (2021)[30].

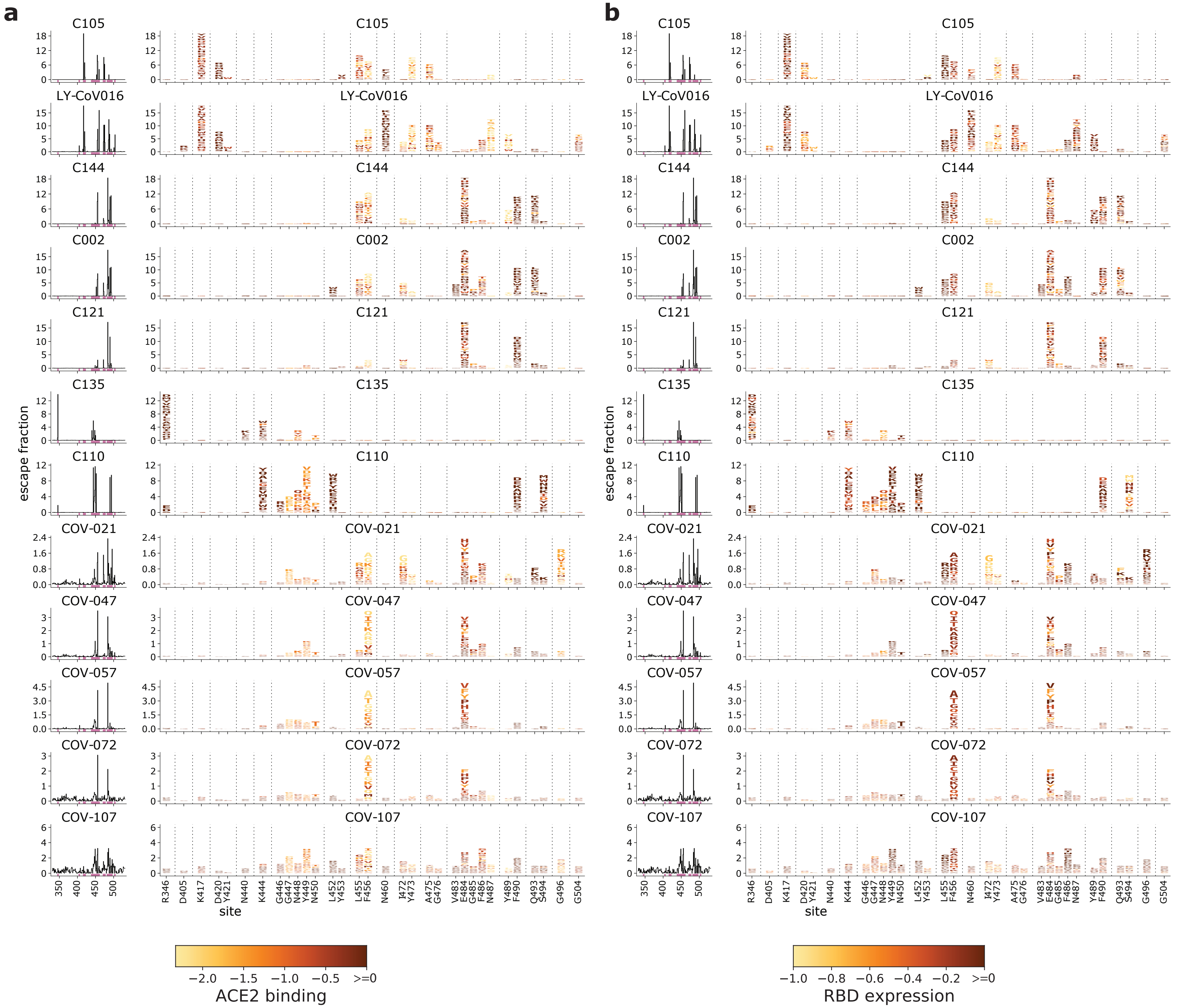


**Figure S4. Logo plots colored according to effects of mutations on ACE2 binding and RBD expression, related to Figures 1, 3, and 4**. Logo plots show the escape fractions of each mutation at key sites (any site called as a site of strong escape for any antibody or plasma). Letters are colored by how mutations affect RBD affinity for ACE2 **(A)** or RBD expression **(B)** as measured via yeast display[32], with yellow indicating poor affinity or expression and brown indicating good affinity and expression. The top 6 rows of logo plots are for monoclonal antibodies; the bottom 5 rows are for polyclonal plasma.

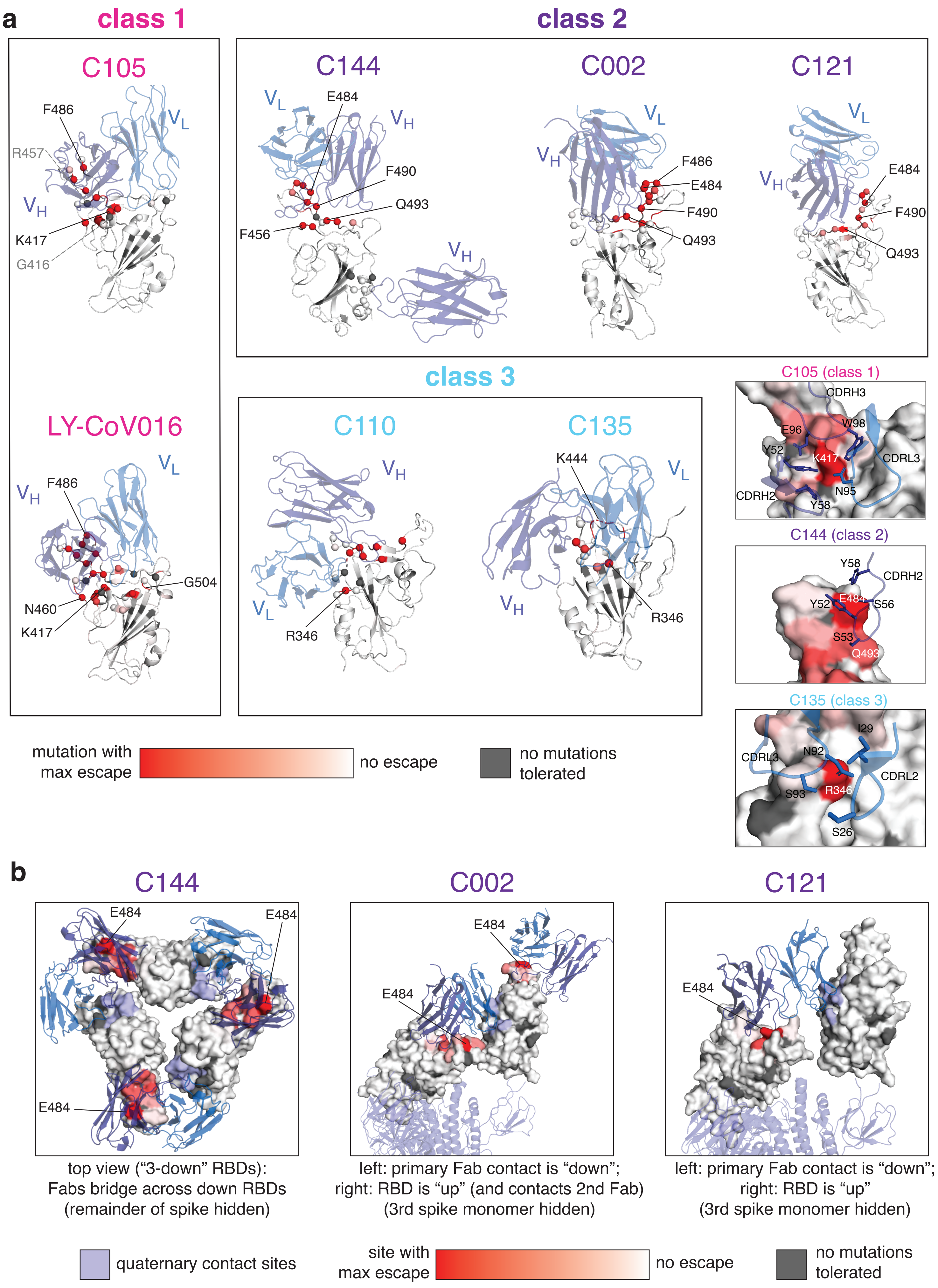
a
class 1
C105
F486
R457
VL
VH
K417
G416
LY-CoV016
VH
F486
VL
G504
N460
K417
class 2
C144
VL
E484
VH
F490
Q493
F456
VH
C002
VL
VH
F486
E484
F490
Q493
C121
VL
VH
E484
F490
Q493
class 3
C110
VH
VL
R346
C135
K444
VL
VH
R346
C105 (class 1)
CDRH3
W98
E96
Y52
K417
CDRL3
N95
CDRH2
Y58
C144 (class 2)
Y58
CDRH2
Y52
E484
S56
S53
Q493
C135 (class 3)
I29
N92
CDRL3
CDRL2
R346
S93
S26
mutation with
max escape
no escape
no mutations
tolerated
b
C144
E484
E484
E484
C002
E484
E484
C121
E484
top view ("3-down" RBDs):
Fabs bridge across down RBDs
(remainder of spike hidden)
left: primary Fab contact is "down";
right: RBD is "up" (and contacts 2nd Fab)
(3rd spike monomer hidden)
left: primary Fab contact is "down";
right: RBD is "up"
(3rd spike monomer hidden)
quaternary contact sites
site with
max escape
no escape
no mutations
tolerated

**Figure S5. Visualization of the maximum escape at a site mapped onto cartoon representations of antibody-bound RBD, related to Figure 2. (A)** Mapping of maximum antibody escape at a site to the antibody-bound RBD. Antibody-contact sites on the RBD (within 4Å) are shown as spheres. Sites with no escape measurements due to excessive functional constraint on the site are shown in dark gray. Each site is colored according to the maximum escape fraction of any mutation at that site (whereas **Figure 2** shows site total escape), scaled from white (no escape) to red (maximum escape for any mutation for that antibody). Inset panels at right indicate key RBD-antibody interactions where mutations to the indicated RBD site disrupt antibody binding. RBD color scale indicates site total escape, as in **Figure 2A**. **(B)** Visualization of class 2 antibody quaternary epitopes. The total escape at each site is mapped onto the surface of the Fab-bound RBD as in **Figure 2A**, with white indicating no escape, and red indicating the site with the most escape from that antibody. Sites where no mutations are tolerated are indicated in dark gray. Antibody quaternary contact sites are shown in periwinkle. The C144 antibody binds to spike trimer in the "all RBDs down" conformation and forms a quaternary epitope that bridges across two adjacent RBDs by binding to a hydrophobic RBD cavity at the base of the N343 N-linked glycan. The C002 and C121 antibodies, when bound to a down-RBD, can form a quaternary epitope with an adjacent up-RBD. The up-RBD also contacts another C121 Fab[25]. Our yeast-display system utilizes monomeric RBD and therefore does not map escape mutations to quaternary contact sites. These results thus cannot be used to determine the importance of the quaternary sites for antibody binding. Previous work, however, has shown that the V367F RBD mutation to the C144 quaternary epitope does not affect neutralization of pseudotyped lentivirus by C144[7]. See **Methods** for PDB accession codes used to generate structural representations. Antibody heavy chains are shown in dark blue, and light chains are in light blue.

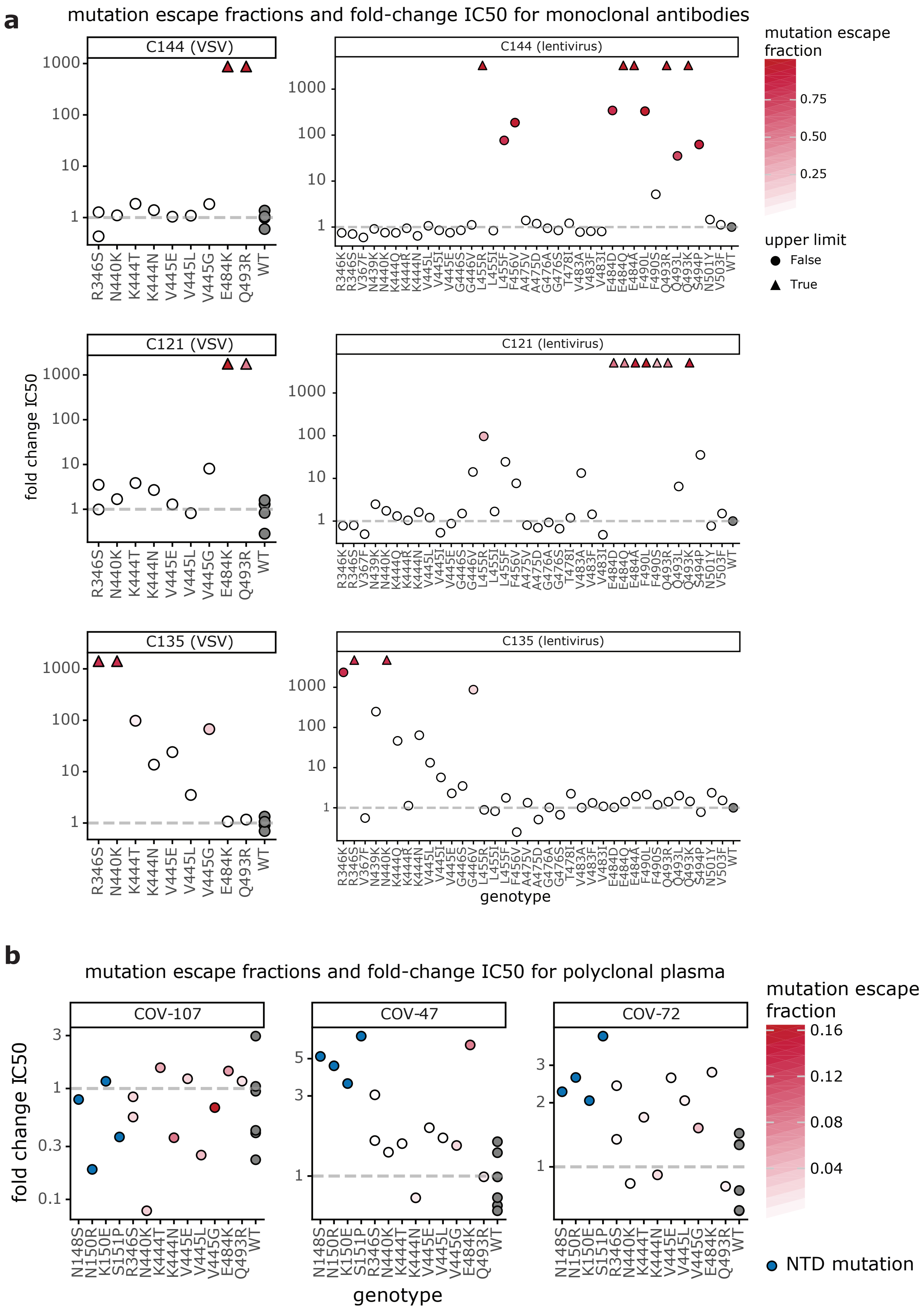
a
mutation escape fractions and fold-change IC50 for monoclonal antibodies
C144 (VSV)
C144 (lentivirus)
C121 (VSV)
C121 (lentivirus)
C135 (VSV)
C135 (lentivirus)
fold change IC50
1
10
100
1000
R346S N440K K444T K444N V445E V445L V445G E484K Q493R WT
R346K R346S V367F N439K N440K K444Q K444R K444N V445L V445I V445E G446S G446V L455R L455I L455F F456V A475V A475D G476A G476S T478I V483A V483F V483I E484D E484Q E484A F490L F490S Q493R Q493L Q493K S494P N501Y V503F WT
genotype
mutation escape fraction
0.75
0.50
0.25
upper limit
False
True
b
mutation escape fractions and fold-change IC50 for polyclonal plasma
COV-107
COV-47
COV-72
fold change IC50
0.1
0.3
1
3
5
2
N148S N150R K150E S151P R346S N440K K444T K444N V445E V445L V445G E484K Q493R WT
genotype
mutation escape fraction
0.16
0.12
0.08
0.04
NTD mutation

**Figure S6. The previously measured effects of spike mutations on neutralization for 3 monoclonal antibodies and 3 polyclonal plasma, related to Figure 4. (A)** The effects of mutations on neutralization of chimeric VSV encoding the SARS-CoV-2 (left) or spike-pseudotyped lentivirus particles (right) by monoclonal antibodies from Weisblum et al. (2020)[7]. The y-axis shows the fold-change in IC50 compared to the Wuhan-Hu-1-like spike, such that larger numbers are greater reductions in neutralization sensitivity. Mutations that had IC50s at or above the limit of detection are indicated as triangles. Points are colored according to their mutation escape fraction (**Figure 1, Supplementary Data 1**). Wildtype is in gray. **(B)** The effects of mutations on neutralization of spike-encoding chimeric VSV by polyclonal antibodies from Weisblum et al. (2020)[7]. Wildtype spike IC50 values are shown in gray. NTD mutations are shown in blue. Escape fraction color scales are independent in **(A)** and **(B)**.

## monoclonal antibody selections

| condition | RBD variant library | number antibody-escaped cells collected | % RBD+ cells in ab-escape gate | estimated RBD+ cells processed | % wildtype cells with 1x [antibody] in ab-escape gate | % wildtype cells with 0.01x [antibody] in ab-escape gate |
|---|---|---|---|---|---|---|
| C135 | 1 | ~2000000 | 16.2 | 12345679 | 0.1 | 94.5 |
| C135 | 2 | 2003895 | 16.8 | 11927946.4 | | |
| C144 | 1 | 1787287 | 15.3 | 11681614.4 | 0 | 94.8 |
| C144 | 2 | 1763435 | 15.5 | 11377000 | | |
| C121 | 1 | 2002999 | 18.4 | 10885864.1 | 0 | 1.2 |
| C121 | 2 | 2013477 | 17.6 | 11440210.2 | | |
| C110 | 1 | 1455039 | 13.5 | 10778066.7 | 0 | 94.8 |
| C110 | 2 | 1458602 | 14.1 | 10344695 | | |
| C105 | 1 | 1603797 | 15 | 10691980 | 0 | 94.5 |
| C105 | 2 | 1600974 | 13.3 | 12037398.5 | | |
| C002 | 1 | 1762675 | 17.1 | 10308040.9 | 0 | 89.5 |
| C002 | 2 | 1740816 | 17.9 | 9725229.05 | | |
| | max | 2013477 | 18.4 | 12345679 | 0.1 | 94.8 |
| | min | 1455039 | 13.3 | 9725229.05 | 0 | 1.2 |

## polyclonal serum selections

| condition | RBD variant library | number antibody-escaped cells collected | % RBD+ cells in ab-escape gate | estimated RBD+ cells processed | % wildtype cells with 1x [serum] in ab-escape gate | % wildtype cells with 0.1x [serum] in ab-escape gate | serum dilution used for library selection |
|---|---|---|---|---|---|---|---|
| COV-021 | 1 | 315013 | 3.1 | 10161709.7 | 0.1 | 6.4 | 1:500 |
| COV-021 | 2 | 443383 | 4.3 | 10311232.6 | | | |
| COV-047 | 1 | 335163 | 3.7 | 9058459.46 | 0 | 0.2 | 1:200 |
| COV-047 | 2 | 358995 | 3.7 | 9702567.57 | | | |
| COV-057 | 1 | 417871 | 4.8 | 8705645.83 | 0 | 5 | 1:50 |
| COV-057 | 2 | 469389 | 4 | 11734725 | | | |
| COV-072 | 1 | 413222 | 2.8 | 14757928.6 | 0 | 11.7 | 1:200 |
| COV-072 | 2 | 374509 | 3 | 12483633.3 | | | |
| COV-107 | 1 | 525925 | 5.1 | 10312254.9 | 0 | 27.2 | 1:80 |
| COV-107 | 2 | 402521 | 2.9 | 13880034.5 | | | |
| | max | 525925 | 5.1 | 14757928.6 | 0.1 | 27.2 | |
| | min | 315013 | 2.8 | 8705645.83 | 0 | 0.2 | |

**Supplementary Table 1. Information on FACS sorting to select cells expressing RBD mutants with reduced binding by antibodies or plasma, related to Figure 1 and Figure S1.**

The file gives the number of antibody-escaped cells collected per selection for each replicate library and the percent of RBD+ cells in the antibody-escape gate for each selection, and the exact dilution used for each plasma selection. The file is also available on GitHub at https://github.com/jbloomlab/SARS-CoV-2-RBD_MAP_Rockefeller/blob/main/data/SupplementaryTable2.xlsx.
