# Supplementary material for: Mapping mutations to the SARS-CoV-2 RBD that escape binding by different classes of antibodies
Source: Nat Commun. 2021 Jul 7;12:4196. doi: 10.1038/s41467-021-24435-8 (PMC8263750; doi:10.1038/s41467-021-24435-8)
Supplement: Supplementary file 3 — Description of Additional Supplementary Files [file 41467_2021_24435_MOESM3_ESM.pdf]

Description of Additional Supplementary Files

File Name: Supplementary Data 1
Description: **Measurements of effects of all amino-acid mutations to the RBD on binding of monoclonal antibodies or polyclonal human plasma, related to Figure** 1. The file gives the escape fraction for each mutation, as well as the total escape fraction at each site and the maximum escape fraction for any mutation at the site. The file is also available on GitHub at https://github.com/jbloomlab/SARS-CoV-2-RBD_MAP_Rockefeller/blob/main/results/supp_data/all_samples_raw_data.csv.
