# Supplementary material for: Mapping mutations to the SARS-CoV-2 RBD that escape binding by different classes of antibodies
Source: Nat Commun. 2021 Jul 7;12:4196. doi: 10.1038/s41467-021-24435-8 (PMC8263750; doi:10.1038/s41467-021-24435-8)
Supplement: Supplementary file 5 — Reporting Summary [file 41467_2021_24435_MOESM5_ESM.pdf]

nature research

Corresponding author(s): Jesse D. Bloom

Last updated by author(s): 6/2/2021

Reporting Summary

Nature Research wishes to improve the reproducibility of the work that we publish. This form provides structure for consistency and transparency in reporting. For further information on Nature Research policies, see our Editorial Policies and the Editorial Policy Checklist.

Statistics

For all statistical analyses, confirm that the following items are present in the figure legend, table legend, main text, or Methods section.

n/a	Confirmed	
☐	☒	The exact sample size (*n*) for each experimental group/condition, given as a discrete number and unit of measurement
☐	☒	A statement on whether measurements were taken from distinct samples or whether the same sample was measured repeatedly
☒	☐	The statistical test(s) used AND whether they are one- or two-sided<br>*Only common tests should be described solely by name; describe more complex techniques in the Methods section.*
☒	☐	A description of all covariates tested
☒	☐	A description of any assumptions or corrections, such as tests of normality and adjustment for multiple comparisons
☐	☒	A full description of the statistical parameters including central tendency (e.g. means) or other basic estimates (e.g. regression coefficient) AND variation (e.g. standard deviation) or associated estimates of uncertainty (e.g. confidence intervals)
☒	☐	For null hypothesis testing, the test statistic (e.g. *F*, *t*, *r*) with confidence intervals, effect sizes, degrees of freedom and *P* value noted<br>*Give P values as exact values whenever suitable.*
☒	☐	For Bayesian analysis, information on the choice of priors and Markov chain Monte Carlo settings
☒	☐	For hierarchical and complex designs, identification of the appropriate level for tests and full reporting of outcomes
☐	☒	Estimates of effect sizes (e.g. Cohen's *d*, Pearson's *r*), indicating how they were calculated

Our web collection on statistics for biologists contains articles on many of the points above.

Software and code

Policy information about availability of computer code

Data collection	During cell sorting for antibody-escaped cells, the FACSDiva software, version 6.1.
Data analysis	The complete computational pipeline for escape-mapping data analysis is available at: https://github.com/jbloomlab/SARS-CoV-2-RBD_MAP_Rockefeller. Markdown summaries of the escape-mapping data analysis steps: https://github.com/jbloomlab/SARS-CoV-2-RBD_MAP_Rockefeller/blob/main/results/summary/summary.md.<br><br>Specifically, we used the dms_variants package (https://jbloomlab.github.io/dms_variants/, version 0.8.5) to process Illumina sequences into counts of each barcoded RBD variant in each pre-sort and antibody-escape population using the barcode/RBD look-up table from Starr et al. (2020). For the analysis in Figure 6, all 765,455 spike sequences on GISAID38 as of May 11, 2021 were downloaded and aligned via mafft, version 7.471. The static logo plots in the paper were created using dmslogo (https://jbloomlab.github.io/dmslogo/) version 0.6.2; a markdown rendering of the code that creates these logo plots is at https://github.com/jbloomlab/SARS-CoV-2-RBD_MAP_Rockefeller/blob/main/results/summary/escape_profiles.md. The interactive visualizations of the escape maps and their projections on the RBD-antibody structures available at https://jbloomlab.github.io/SARS-CoV-2-RBD_MAP_Rockefeller/ were created using dms-view (https://dms-view.github.io/docs/). In Figure 5C and Figure S6, the numerical IC50 values were extracted from figures in Weisblum et al. (2020) using the WebPlotDigitizer tool v4.4 (https://apps.automeris.io/wpd/). The multidimensional scaling in Figure 1C and Figure 4A that projects the antibodies into a two-dimensional space of escape mutations was performed using the Python scikit-learn package, version 0.23.2. Antibody-RBD structural contacts were annotated using the bio3d package, version 2.4.0, in R version 3.6.2.

For manuscripts utilizing custom algorithms or software that are central to the research but not yet described in published literature, software must be made available to editors and reviewers. We strongly encourage code deposition in a community repository (e.g. GitHub). See the Nature Research guidelines for submitting code & software for further information.

Data

Policy information about availability of data

All manuscripts must include a data availability statement. This statement should provide the following information, where applicable:

- Accession codes, unique identifiers, or web links for publicly available datasets
- A list of figures that have associated raw data
- A description of any restrictions on data availability

All data in the manuscript are available without restriction.
Raw data tables of mutant escape fractions are in Supplementary Table 1: https://github.com/jbloomlab/SARS-CoV-2-RBD_MAP_Rockefeller/blob/main/results/supp_data/all_samples_raw_data.csv. These data are the escape fractions shown in Figures 1-6 and Figures S2-S6.
Raw Illumina sequencing for the escape mapping: NCBI SRA, BioProject: PRJNA639956, BioSample SAMN18148595.
Processed Illumina sequencing counts for the escape mapping: https://github.com/jbloomlab/SARS-CoV-2-RBD_MAP_Rockefeller/blob/main/results/counts/variant_counts.csv
The following publicly available datasets were used in the analysis in this study:
The PBD accessions for the antibody-S complex structures are: 6XCM and 6XCN for C105, 7C01 for LY-CoV016, 7K8S and 7K8T for C002, 7K8X and 7K8Y for C121, 7K90 for C144, 7K8Z for C135, and 7K8V for C11025,28,29. The protein sequences for these antibodies are available at the aforementioned PDB accession numbers.
Surface representations of the RBD for non-antibody-bound structures utilize PDB 6M0J65.
The “wildtype” SARS-CoV-2 RBD sequence is that from isolate Wuhan-Hu-1 (Genbank accession number MN908947, residues N331-T531).
The analysis in Figure 6 uses all 765,455 spike sequences on the GISAID EpiCoV database (https://www.gisaid.org/) as of May 11, 2021.

Together, these are the minimal set of data required to replicate the analysis.

Field-specific reporting

Please select the one below that is the best fit for your research. If you are not sure, read the appropriate sections before making your selection.

☒ Life sciences ☐ Behavioural & social sciences ☐ Ecological, evolutionary & environmental sciences

For a reference copy of the document with all sections, see nature.com/documents/nr-reporting-summary-flat.pdf

Life sciences study design

All studies must disclose on these points even when the disclosure is negative.

Sample size	No sample size calculations were performed. n=2 independent, biological replicate libraries were used for all escape-mapping experiments. The mutation- and site-level escape metrics were well-correlated between the two independent libraries, so biological duplicate libraries were determined to be sufficient. The numbers of antibodies and plasma samples used in the study were determined by the number of available monoclonal antibodies and polyclonal plasma samples from individuals previously infected with SARS-CoV-2.
Data exclusions	No data were excluded from analyses.
Replication	n=2 independent, biological replicate SARS-CoV-2 RBD deep mutational scanning libraries were used for all escape-mapping experiments. All attempts at replication were successful and are reported in the manuscript.
Randomization	For no analyses did we determine statistical significance of results. No randomization, covariate analyses, or statistical tests were performed. We did not determine statistical significance for the escape metrics as there is no established method for doing so.<br>In the Methods, we report here the details of quantitative analysis of the effects of mutations on antibody binding to the RBD.<br>Samples were not assigned to different cohorts or study arms, so randomization would not apply to the work done in this study. All samples were treated the same.
Blinding	No group allocation or blinding was performed in this study.

Reporting for specific materials, systems and methods

We require information from authors about some types of materials, experimental systems and methods used in many studies. Here, indicate whether each material, system or method listed is relevant to your study. If you are not sure if a list item applies to your research, read the appropriate section before selecting a response.

Materials & experimental systems

n/a	Involved in the study
☐	☒ Antibodies
☒	☐ Eukaryotic cell lines
☒	☐ Palaeontology and archaeology
☒	☐ Animals and other organisms
☐	☒ Human research participants
☒	☐ Clinical data
☒	☐ Dual use research of concern

Methods

n/a	Involved in the study
☒	☐ ChIP-seq
☐	☒ Flow cytometry
☒	☐ MRI-based neuroimaging

Antibodies

Antibodies used	C105, LY-CoV016, C121, C144, C002, C110, C135 monoclonal antibodies were isolated from SARS-CoV-2 convalescent individuals as described by Robbiani, et al. (2020) and Shi et al. (2020). These antibodies all bind to the SARS-CoV-2 receptor-binding domain (RBD). Escape-mapping experiments also used the following secondary antibodies: PE-conjugated goat anti-human-IgG (Jackson ImmunoResearch 109-115-098) to label for bound monoclonal antibody or Alexa-647-conjugated goat anti-human-IgA+IgG+IgM (Jackson ImmunoResearch 109-605-064) to label for bound plasma antibodies, and FITC-conjugated anti-Myc (Immunology Consultants Lab, CYMC-45F) to label for RBD surface expression. For this antibody, the manufacturer website (https://www.icllab.com/anti-c-myc-antibody-chicken-fitc-13661.html) states: "This antibody will react with EQKLISEEDL as determined by ELISA and IP techniques." This antibody was validated for binding to yeast-displayed myc-tagged proteins in Procko et al. (2013) (doi 10.1016/j.jmb.2013.06.035) and validated for binding to yeast-displayed myc-tagged RBD in Starr et al. (2020) (doi:10.1016/j.cell.2020.08.012).
Validation	These antibodies were previously structurally characterized in complex with SARS-CoV-2 S trimer in Barnes, et al. (2020a), Barnes, et al., (2020b), and Shi et al. (2020). The neutralization properties of C135, C144, and C121 were functionally characterized in Weisblum et al. (2020). The PBD accessions for the antibody-S complex structures are: 6XCM and 6XCN for C105, 7C01 for LY-CoV016, 7K8S and 7K8T for C002, 7K8X and 7K8Y for C121, 7K90 for C144, 7K8Z for C135, and 7K8V for C110.

Human research participants

Policy information about studies involving human research participants

Population characteristics	No covariate analyses were performed in this study. Plasma samples were previously described and collected as part of a prospective longitudinal cohort study of SARS-CoV-2 convalescent adult individuals in New York, NY (Robbiani et al (2020)). Plasma samples profiled in this study were obtained 21-35 days post-symptom onset.
Recruitment	Plasma samples were previously described and collected as part of a prospective longitudinal cohort study of SARS-CoV-2 convalescent individuals in the tristate region near New York, NY (Robbiani et al (2020)). Plasma samples profiled in this study were obtained 21-35 days post-symptom onset. According to Robbiani et al: "Study participants were recruited at the Rockefeller University Hospital in New York from 1 April to 8 May 2020. Eligible participants were adults aged 18–76 years who were either diagnosed with a SARS-CoV-2 infection by RT–PCR and were free of symptoms of COVID-19 for at least 14 days (cases), or who were close contacts (for example, household members, co-workers or members of same religious community) of someone who had been diagnosed with a SARS-CoV-2 infection by RT–PCR and were free of symptoms suggestive of COVID-19 for at least 14 days (contacts). Exclusion criteria included the presence of symptoms suggestive of an active SARS-CoV-2 infection, or haemoglobin levels of <12 g/dl for men and <11 g/dl for women." Therefore, self-selection bias could have influenced whether participants chose to enroll in the study or not. For instance, participants must have been willing and able to travel to the Rockefeller University Hospital and to interact with healthcare personnel near the beginning of the COVID-19 epidemic in the region. The study was also limited to the tri-state region and may not be representative of other geographic locations.
Ethics oversight	Samples were obtained upon written consent from community participants under protocols approved by the Institutional Review Board of the Rockefeller University (DRO-1006).

Note that full information on the approval of the study protocol must also be provided in the manuscript.

Flow Cytometry

Plots

Confirm that:

☒ The axis labels state the marker and fluorochrome used (e.g. CD4-FITC).

☒ The axis scales are clearly visible. Include numbers along axes only for bottom left plot of group (a 'group' is an analysis of identical markers).

☒ All plots are contour plots with outliers or pseudocolor plots.

☒ A numerical value for number of cells or percentage (with statistics) is provided.

Methodology

Sample preparation	Antibody labeling and selection was performed essentially as described in Greaney, et al. (2020)30. Specifically, 9 OD aliquots of RBD libraries were thawed and grown overnight at 30°C 275 rpm in 45mL SD-CAA (6.7 g/L Yeast Nitrogen Base, 5.0 g/L Casamino acids, 1.065 g/L MES, and 2% w/v dextrose). Libraries were diluted to an OD of 0.67 in SG-CAA+0.1% dextrose (SD-CAA with 2% w/v galactose and 0.1% w/v dextrose in place of 2% dextrose), and incubated for 16-18 hours at room temperature with mild agitation to induce RBD surface expression. For each antibody selection, 20 OD units of induced cells were washed twice with PBS-BSA (0.2 mg/mL), and incubated in 4mL of PBS-BSA with monoclonal antibody or plasma for 1 h at room temperature with gentle agitation. Incubations were performed with 400 ng/mL for each monoclonal antibody (C105, C144, C002, C121, C135, or C110) or with a sub-saturating dilution of polyclonal plasma such that the amount of fluorescent signal due to plasma antibody binding to RBD was approximately equal across plasma (COV-021, 1:500; COV-047, 1:200; COV-057, 1:50; COV-072, 1:200; COV-107, 1:80). Labeled cells were washed with ice-cold PBS-BSA followed by secondary labeling for 1 h at 4°C in 2.5 mL 1:200 PE-conjugated goat anti-human-IgG (Jackson ImmunoResearch 109-115-098) to label for bound monoclonal antibody or 1:200 Alexa-647-conjugated goat anti-human-IgA+IgG+IgM (Jackson ImmunoResearch 109-605-064) to label for bound plasma antibodies, and 1:100 FITC-conjugated anti-Myc (Immunology Consultants Lab, CYMC-45F) to label for RBD surface expression. Labeled cells were washed twice with PBS-BSA and resuspended in 2.5mL PBS. Yeast expressing the unmutated SARS-CoV-2 RBD were prepared in parallel to library samples, labeled at the same 400 ng/mL and 100x reduced 4 ng/mL antibody concentrations for the monoclonal antibodies, and with 1x and 10x reduced plasma concentrations for the polyclonal plasma.
Instrument	Yeast cells expressing RBD variants with substantially reduced antibody binding were selected via fluorescence-activated cell sorting (FACS) on a BD FACSAria II.
Software	FACSDiva software was used to set selection gates during cell sorting. No analysis was performed on the flow cytometry data itself. The complete computational pipeline for escape-mapping data analysis is available at https://github.com/jbloomlab/SARS-CoV-2-RBD_MAP_Rockefeller.
Cell population abundance	A nested FACS gating strategy was used for all antibody- and plasma-escape selection experiments to select for single cells (SSC-A vs. FSC-A, SSC-W vs. SSC-H, and FSC-W vs. FSC-H) that also express RBD (FITC-A vs. FSC-A). Then, cells with substantially reduced antibody binding were selected on a 2D scatter plot with RBD expression on the x-axis and antibody binding on the y-axis. For monoclonal antibody selections, FACS selection gates were drawn to capture 95% of yeast expressing unmutated SARS-CoV-2 RBD labeled at 100x reduced antibody concentration relative to library samples. For polyclonal plasma selections, FACS selection gates were drawn to capture 2.8–5% of the RBD mutants with the lowest amount of plasma binding for their degree of RBD expression. Specifically, the fractions of RBD+ cells that fell into the antibody-escape bin for each antibody (prefixed with C*) or plasma (prefixed with COV*) and each biological replicate library were as follows: C135: 16.2 and 16.8%; C144: 15.3 and 15.5%; C121: 18.4 and 17.6%; C110: 13.5 and 14.1%; C002: 17.1 and 17.9%; COV-21: 3.1 and 4.3%; COV-47: 3.7 and 3.7%; COV-57: 4.8 and 4%; COV-72: 2.8 and 3%; COV-107: 5.1 and 2.9%. Nearly zero (<0.1%) and 0.2 to 27.2% of cells expressing unmutated RBD fell into this gate when stained with 1x and 0.1x the concentration of plasma, respectively. For each sample, approximately 10 million RBD+ cells (range 8.7e6 to 1.5e7 cells) were processed on the cytometer, with between 1.5e6 and 2.0e6 monoclonal antibody-escaped cells and 3.2e5 and 5.3e5 plasma-escaped cells collected per sample. These numbers are also listed in Supplementary Table 1.
Gating strategy	Yeast cells expressing RBD variants with substantially reduced antibody binding were selected via fluorescence-activated cell sorting (FACS) on a BD FACSAria II. For monoclonal antibody selections, FACS selection gates were drawn to capture 95% of yeast expressing unmutated SARS-CoV-2 RBD labeled at 100x reduced antibody concentration relative to library samples. For polyclonal plasma selections, FACS selection gates were drawn to capture 2.8–5% of the RBD mutants with the lowest amount of plasma binding for their degree of RBD expression (Figure S1A-C). Nearly zero (<0.1%) and 0.2 to 27.2% of cells expressing unmutated RBD fell into this gate when stained with 1x and 0.1x the concentration of plasma, respectively. For each sample, approximately 10 million RBD+ cells (range 8.7e6 to 1.5e7 cells) were processed on the cytometer, with between 1.5e6 and 2.0e6 monoclonal antibody-escaped cells and 3.2e5 and 5.3e5 plasma-escaped cells collected per sample. Antibody-escaped cells collected per sample into SD-CAA supplemented with 1% w/v BSA and grown overnight in 1.5mL SD-CAA + 100 U/mL penicillin + 100 µg/mL streptomycin at 30°C 275 rpm.

☒ Tick this box to confirm that a figure exemplifying the gating strategy is provided in the Supplementary Information.
