# Supplementary material for: Mapping mutations to the SARS-CoV-2 RBD that escape binding by different classes of antibodies
Source: Nat Commun. 2021 Jul 7;12:4196. doi: 10.1038/s41467-021-24435-8 (PMC8263750; doi:10.1038/s41467-021-24435-8)
Supplement: Supplementary file 2 — Peer Review File [file 41467_2021_24435_MOESM2_ESM.pdf]

Editorial Note: This manuscript has been previously reviewed at another journal that is not operating a transparent peer review scheme. The manuscript was considered suitable for publication without further review at Nature Communications.
